# Hyphal Growth in Human Fungal Pathogens and Its Role in Virulence

**DOI:** 10.1155/2012/517529

**Published:** 2011-11-09

**Authors:** Alexandra Brand

**Affiliations:** School of Medical Sciences, University of Aberdeen, Institute of Medical Sciences, Foresterhill, Aberdeen AB25 2ZD, UK

## Abstract

Most of the fungal species that infect humans can grow in more than one morphological form but only a subset of pathogens produce filamentous hyphae during the infection process. This subset is phylogenetically unrelated and includes the commonly carried yeasts, *Candida albicans*, *C. dubliniensis*, and *Malassezia* spp., and the acquired pathogens, *Aspergillus fumigatus* and dermatophytes such as *Trichophyton rubrum* and *T. mentagrophytes*. The primary function of hypha formation in these opportunistic pathogens is to invade the substrate they are adhered to, whether biotic or abiotic, but other functions include the directional translocation between host environments, consolidation of the colony, nutrient acquisition and the formation of 3-dimensional matrices. To support these functions, polarised hyphal growth is co-regulated with other factors that are essential for normal hypha function *in vivo*.

## 1. Introduction

The commonly carried yeasts, *Candida albicans*, *C. dubliniensis*, and *Malassezia* spp., and the acquired pathogens, *Aspergillus fumigatus *and the dermatophytes are opportunistic pathogens and afflict individuals whose immune systems are compromised or dysfunctional, often in combination with other predisposing factors. *Candida* spp. are regarded as part of the commensal flora, colonising multiple mucosal sites, including the GI tract. They can cause irritating superficial oral and vaginal infections “thrush”, invasion of the skin and nails (onychomycosis), and, if released into the bloodstream of immunocompromised patients, fatal systemic infections due to the formation of inflamed lesions in internal organs. *Malassezia* spp. are also carried by the majority of the population. All but one species lacks fatty acid synthase activity, so *Malassezia* localise to epidermal surfaces that are rich in secreted sebum, such as the scalp, chest, and back [1–3]. *Malassezia* cause a variety of superficial fungal infections, including seborrhoeic dermatitis, dandruff, *Tinea versicolor*, atopic dermatitis, and psoriasis, and often exacerbate other skin conditions. *Aspergillus* spp. are obligately filamentous and grow as mycelia in the soil. They produce microscopic airborne conidia which are inhaled into the lung, from where they are normally cleared by the activity of airway cilia and alveolar macrophages. Failure to do so can lead to several diseases, including invasive aspergillosis, where the organism is carried in the blood to other organs, and aspergilloma, the formation of fungal foci in cavities formed in a prediseased lung. The obligate hyphal growth of *Aspergillus*, where little or no cell shedding occurs, is a contributing factor to morbidity due to the potential delay in diagnosing bloodstream infection [4]. The attributable mortality rate for *Aspergillus* spp. is 50–80%, the highest rate for fungal infections in humans [5]. Dermatophytes are acquired from the environment through passage from mammalian reservoirs and between humans. Approximately one-third of the European population is estimated to suffer from acute dermatophyte infections such as *Tinea pedis* “athlete's foot” through their ability, shared with *Candida* spp., to degrade heavily keratinised skin and thrive in warm, moist conditions [6]. A further threat posed to health by these filament-forming pathogens is their propensity to develop pseudomembranous biofilms. *C. albicans* biofilms appear as thick, creamy-white plaques on mucosal surfaces, the typical manifestation of thrush. Aspergilloma foci in the lung display many of the characteristics associated with biofilms. *C. albicans*, *Malassezia*, and *A. fumigatus* are also found in biofilms on abiotic medical plastics. The biofilm environment poses significant medical problems since it promotes resistance to antifungal drugs, acts as a reservoir for seeding further infection, and compromises the mechanical function of devices such as voice prostheses and contact lenses [7, 8]. 

Many of the fungi that cause disease in humans grow as free-living mycelia in the environment but convert to a yeast morphology once inside the human body in response to the raise in temperature. Collectively known as “the dimorphs,” this group is able to cause severe and sometimes fatal infection whilst existing solely in the yeast form, illustrating that hypha formation *per se* is not essential for fungal virulence. Microscopy of diseased tissue show that *C. albicans*, *Malassezia*, and dermatophytes reversibly switch between yeast and hyphal morphologies *in vivo*. When reverse genetics became available in *C. albicans* nearly two decades ago, it was shown that loss of the ability to make this transition resulted in avirulence [9]. The fungi most associated with morbidity and mortality in humans, therefore, require a morphogenesis programme for full virulence, underlining the need to understand the temporal and spatial role of morphogenesis during disease progression. The challenge has been partially met by the development of genetic tools and infection models for *C. albicans*, *C. dubliniensis*, and* A. fumigatus*, where there is inherent difficulty in studying the development of infection *in vivo*. Less emphasis has been placed on the study of the *Malassezia *and dermatophytes because they cause only superficial infections and are more amenable to topical treatment. Nevertheless, such infections are extremely common and can cause considerable distress to chronic sufferers. Reverse genetics approaches and genome sequencing data are now becoming available for these organisms and highlight the contrasts and commonalities with the more well-studied pathogens [2, 10–12].

We are gaining a better understanding of the role of hyphae in disease and appreciate that hyphae come as a complex package of morphological features, supported by hypha-specific, site-specific, and time-dependent gene regulation. The bottom line, perhaps not surprisingly, is that hypha formation confers the ability to actively penetrate host tissue. However, there is still much to learn about how hyphae are deployed effectively during tissue translocation, adherence and nutrient acquisition *in vivo*. The regulation of morphogenesis in *C. albicans* and models of hyphal growth are covered in excellent reviews elsewhere [18–24]. This paper focuses on presenting what is known about hyphal growth during infection by the major hypha-forming human pathogens and considers the specific functions and mechanical and structural properties that hyphae confer during virulent fungal growth.

## 2. Hyphal Growth in Disease

Fungi infect a diverse range of host body sites. *Malassezia *and the dermatophytes are limited to specific environments partially due to nutrient availability, but *C. albicans* and *A. fumigatus* can tolerate high internal body temperatures and a wide range of ambient pH values, oxygen availability, and nutrients. Each environment presents challenges that select for fungi with specific adaptations. 

### 2.1. Keratinised Cell Layers

**Figure 1:**
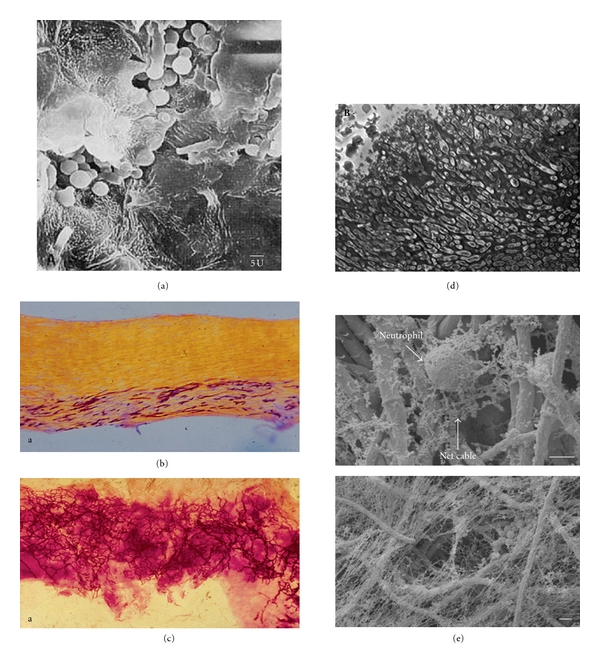
*In vivo* growth of filamentous fungal pathogens. (a) Hyphae of *Malassezia globosa *translocate deeper within the keratinised epidermal layer where they establish new colonies and revert to growth as yeast [13]. (b) Dermatophyte hyphae follow the keratinised layers that run parallel to the nail surface [14]. (c) Hyphae of *Candida albicans* growing in a multidirectional manner within the nail, often forming helical twists [14]. (d) Histological section of aspergilloma in the lung showing the tightly packed *Aspergillus fumigatus* hyphae surrounded by a matrix material and with no immune-cell infiltrate [15]. (e) Production of neutrophil extracellular traps (NETs) against *A. fumigatus* hyphae where, unlike the safe haven of a fungus-derived biofilm matrix, hyphae are instead imprisoned in a host-derived matrix of neutrophil DNA and calprotectin, a protein which chelates the divalent cations that are required for fungal growth [16, 17].

The epidermis is made up of dense, heavily keratinised cell layers that are constantly undergoing a process of renewal. Three major groups of filament-forming fungi commonly infect the keratinised and cornified layers of skin, nails, and hair—*Candida albicans*, the dermatophytes and the lipophilic *Malassezia* spp. Conidia and hyphae are observed skin scrapings and in lesions caused by these fungi, which face the challenge of retaining a foothold on a surface that is being constantly shed. *Candida* spp. and *Malassezia* spp. are part of the commensal flora and growth in the yeast form is generally asymptomatic and tolerated by the immune system. Although phylogenetically distant, these fungi have evolved a similar arsenal of lipases and proteases for life on humans, some of which are specifically expressed during growth as hyphae [2, 25]. *Malassezia* yeasts are thought to be taken up by keratinocytes and exist as facultative intracellular parasites by actively suppressing the inflammatory response [26]. *Malassezia* hyphae are not well studied because they are slow growing *in vitro* and require specialised growth media that contains a source of lipid. *In vivo*, hyphae are observed only in individuals with hyperactive sebaceous-gland activity, where the presence of excess sebum appears to be the inducer of morphogenesis [27]. *M. globosa* grows as yeast and hyphae in localised cavities formed within the rough landscape of the skin. The hyphae are short but penetrate keratinised skin cells to gain access to deeper cavities below, where growth reverts to yeast, and new colonies are formed (Figure 1(a)) [13, 14]. One of the roles of *M. globosa* hyphae, therefore, appears to be the rerooting of infection in nutrient-rich, deep cornified layers to replace the older fungal colonies that are passively brought to the epidermal surface and sloughed off. In lipophilic *Malassezia *spp., morphogenesis is thought to be a response to the presence of excess sebum [28]. In *M. globosa*, fungal lipases break down sebaceous triglycerides, producing abundant unsaturated fatty acids as unwanted byproducts, which act as immunostimulatory molecules in individuals with poor epidermal integrity [3, 29, 30]. Eleven protein allergens have been identified from *Malassezia* species, including a conserved Heat Shock Protein (HSP70) [31]. Interestingly, the *C. albicans* surface invasin, Ssa1, is also an HSP70-like protein [32, 33]. The specific involvement of hyphae in the generation of immunostimulatory molecules by *Malassezia* has yet to be addressed.

The keratinolytic dermatophytes,* T. mentagrophytes* and *Arthroderma benhamiae*, are acquired from other mammalian hosts and thrive in warm, damp skin [11]. Dermatophytes are visible as arthroconidia and hyphae within the epidermal layers and surrounding hair shafts, although the *in situ* signals that induce morphogenesis are not known. In addition to hyphae, *T. mentagrophytes* cells produce thin fibrils, proposed to be adhesin molecules, which help attach the fungus to the skin surface [28]. The deeper layers of the epidermis are penetrated by hyphae that meander and produce branches that extend parallel to the predominant cell layers (Figure 1(b)). It is thought that further penetration is inhibited by limited iron availability due to the activity of host ferritin in the underlying layer of the dermis [34]. No direct penetration of the keratinised cells by dermatophytes has been reported, but fungal cells flatten themselves against the substratum, a further possible aid to adhesion [28]. In contrast, *C. albicans* growth in the nail showed a more chaotic pattern of colonisation. Hyphae were entangled and less likely to stratify within the epidermal layers than dermatophytes or *Malassezia* species [14]. Additionally, many hyphae grew in a helical or spiral growth trajectory, a contact-dependent and low-nutrient response in this fungus (Figure 1(c)) [35, 36]. Detection of an epidermal infection by the host leads to the proliferation of epidermal cells to increase shedding of the stratum corneum and the microbes contained within it. The physical penetration by hyphae and their accompanying enzymatic activity, therefore, contribute to the thickened and chaotic appearance of the skin and nail bed that is a marked feature of epidermal infection. 

### 2.2. Biofilms on Mucosal and Abiotic Surfaces

**Figure 2:**
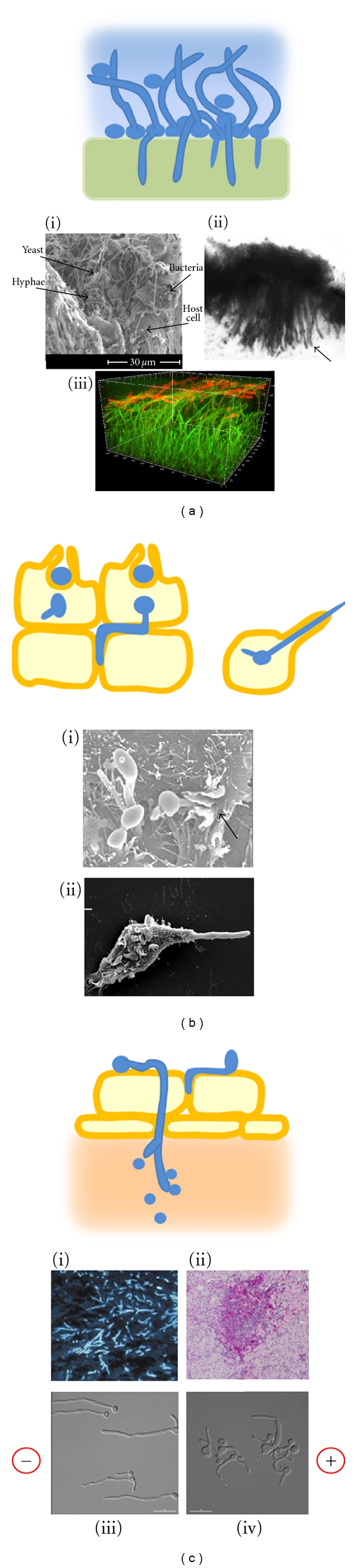
Models and examples of hyphal invasion *in vivo*. (a) Biofilms form on mucosal and abiotic surfaces by initial adhesion of yeast cells, followed by hypha germination and the deposition of extracellular matrix polysaccharides (blue). On mucosa or soft silicones, hyphae penetrate the underlying layers. (i) Rat denture biofilm formation after 48 h [37]. (ii) Hyphae penetrate the silicone of a voice prosthesis, causing it to expand and stiffen [7]. (iii) *In silico* construction of a biofilm showing the thick hyphal matrix (green) and leading edge of *β*-glucan deposition at the hyphal tips (red) [38]. (b) Induced uptake of yeast and newly germinating hyphae by epithelial cells or phagocytosis by macrophages is followed by sustained polarised growth, which breaches the host cell plasma membrane and permits the escape of the fungus. (i) *C. albicans* hyphae are engulfed by epithelial cells during induced uptake [39]. (ii) *C. albicans* avoids being killed by a macrophage by undergoing morphogenesis and breaching the macrophage membrane [40]. (c) Active penetration of endothelial cells and reversion to yeast growth in the tissue below. (i) Biopsy of murine lung with invasive aspergillosis, showing septate hyphae stained with blancophor [41]. (ii) Histological section of murine kidney showing *C. albicans* lesion containing yeast, hyphae, and infiltrate of neutrophils (courtesy D. MacCallum). (iii) Galvanotropism: *C. albicans* wild-type hyphae orient towards the cathode when grown in an applied electric field (10 V/cm) [42]. (iv) All tropic responses were abolished in the *rsr1*Δ mutant, which was attenuated in virulence. Cell polarity was maintained, but hyphal tip directionality was erratic [42].

The formation of hyphae is a key feature in the development of the 3-dimensional structure of fungal biofilms, which present specific clinical problems due to their relative resistance to treatment with antifungal drugs and their potential to release infective cells. Biofilms formed by *C. albicans* are the primary cause of mucosal infection in susceptible humans, where the plaques formed by overproliferation of adhered cells to vaginal or oral epithelia are easily visible [38, 43]. Surprisingly little is known about the activation of morphogenesis by *C. albicans* during mucosal infection, although predisposing factors such as gender, age, poor oral hygiene, and underlying chronic conditions are well-documented. In *in vitro* models of epithelial infection, the germination of hyphae commences as soon as yeast cells are introduced into the system [44]. This begs the question as to whether this process is actively suppressed during commensalism, or whether hyphae are constantly germinating *in vivo* but immediately being cleared by the immune system. Contaminated medical plastics, such as catheters and prostheses, also offer stable substrates for biofilm formation (Figure 2(a)(i)), and recent studies of aspergilloma suggest that these enclosed foci of matrix-bound *A. fumigatus* hyphae found in the diseased lung also display the characteristic properties of biofilms (Figure 1(d)) [15, 45]. *In vitro* studies of the temporal process of biofilm formation by *C. albicans* on inert, abiotic substrates show that yeast cells provide an early-stage, adhesive base layer, from which hyphae germinate to generate a thick, loosely structured matrix (Figure 2(a)(iii)). As the biofilm matures, the matrix becomes embedded through the production of fungal exopolysaccharides (*β*-glucan), protein, hexosamine and extracellular DNA [46, 47]. Hypha formation is not essential for biofilm establishment or maintenance, but biofilms formed by yeast alone are thin and more easily removed from surfaces by mechanical disruption, suggesting that the tangle of hyphal filaments serves to strengthen the structure [48]. Studies of *C. albicans* biofilms formed on mucosal tissue showed that this structure is retained *in vivo*, but the matrix is more complex, comprising yeast, hyphae, extracellular *β*-glucan, bacteria, keratinised squamous cells, and foci of host neutrophils [38]. 

Hyphae in mature biofilms show a strong propensity to invade the underlying substrate, even when there is little nutrient value (Figure 2(a)). In the mucosa, intercalation of hyphae disrupts the epithelial layer, which activates a localised inflammatory response. Remarkably, the hyphae of *C. albicans* and a variety of other filamentous pathogens are able to penetrate the soft medical silicones which are used in the manufacture of prosthetic devices and contact lenses. Hyphal infiltration causes the silicone to expand and stiffen, thus, compromising the function of the device (Figure 2(a)(ii)) [7, 8]. Mucosal biofilms form at sites occupied by multiple species of microbes [49]. The presence of bacteria within *C. albicans* biofilms is, therefore, not surprising, and studies suggest that the growth of hyphae within biofilms is likely to be modulated *in vivo* by species such as *Streptococcus mutans* and *Streptococcus gordonii*. These oral bacteria can attenuate or promote hyphal growth, respectively, through physical interactions and chemical signalling via the production of quorum-sensing molecules [50, 51]. *C. albicans* produces its own quorum-sensing molecules, farnesol, and tyresol, which are negative and positive regulators of morphogenesis, respectively, [52–54]. The role of hyphae in mucosal infection by *C. albicans* is, therefore, modulated by a three-way interaction between the fungus, resident bacteria and the ambient host environment.

### 2.3. Systemic Dissemination

*A. fumigatus* and *C. albicans* both cause bloodstream infections in humans (invasive aspergillosis and disseminated candiasis) and employ two primary methods by which to translocate from one environment to another within the body. The first method is through the passive uptake of fungal particles by host cells, and the second is by a method that is unique to filamentous fungi-active penetration of host cell membranes by the hyphal tip [55]. Passive uptake occurs either during receptor-mediated engulfment of the fungus by phagocytic cells with the aim of killing the microbe or by nonphagocytic endothelial or epithelial cells where molecules on the fungal surface stimulate their own endocytosis (Figure 2(b)) [40, 56, 57]. *C. albicans* and *A. fumigatus* both appear to use this method early in the infection process, translocating by induced uptake into superficial epithelial cells or the endothelial cells that line blood vessels. It has been observed that germinating *C. albicans* hyphae are more efficiently endocytosed than yeast cells [58]. This could be the result of evolutionary pressure on the fungus because the immune system is activated by the presence of hyphae, making escape into a safer environment a matter of some urgency for the fungus. This is of particular importance in the bloodstream because exposure to serum is a strong inducer of morphogenesis in *C. albicans*. The molecular interactions that stimulate the uptake of *C. albicans* by nonphagocytic cells are being elucidated and involve the expression of fungal surface invasins, Als3 and Ssa1. These proteins have alternative cellular functions (Als3 is an amyloid-like, hypha-specific adhesion, and Ssa1 is an intracellular heat-shock protein), but Ssa1 interacts with N-cadherin on endothelial cells and both interact with E-cadherin on epithelial cells [33, 57, 59]. *In vitro* assays show that *A. fumigatus* conidia may translocate from the lung alveoli by induced uptake into Type II alveolar epithelial cells, which display immune cell activity [4, 60, 61]. Escape from the intracellular host environment requires morphogenesis followed by sustained polarised growth. *C. albicans* mutant cells with deletion of Eed1, which is involved in polarity maintenance, could penetrate epithelial cells during the onset of hyphal growth (Figure 2(b)(i)) but became trapped intracellularly when polarity could not be maintained [39]. As growth reverted to yeast, the mutant was unable to punch its way out of the host cell, and hence, dissemination into the underlying cell layers did not occur. Sustained hyphal growth is, therefore, important for the endocytic route of tissue invasion in *C. albicans*, and Eed1 may also be required for escape from host macrophages after phagocytosis (Figure 2(b)(ii)).

This finding underscores a fundamental difference between the mechanisms used by pathogens that undergo morphogenesis and those that infect solely as yeast, such as *Cryptococcus neoformans*. The dissemination of *C. neoformans* from the lung also occurs via induced uptake, but the organism has an alternative way of escaping from the host cell. Unlike *C. albicans*, it is able to modify the intracellular environment of the host cell and proliferate until it finally causes host-cell lysis or is expelled from it by nonlytic exocytosis [62, 63]. Induced uptake appears to be a common strategy for the first step of translocation within the host, but pathogenic fungi have alternative mechanisms for breaking free from the host vehicle. In hypha-generating fungi, the second stage of invasion is one of morphogenesis followed by the active penetration of host cell membranes by hyphal tips (Figure 2(c)). This enables the fungus to establish fungal masses in the underlying matrix of solid organ tissue where, compared to the bloodstream, infiltrates of host immune cells find it relatively difficult to access the invading fungus (Figures 2(c)(i) and 2(c)(ii)). 

## 3. The Physical Properties of Hyphae

### 3.1. Morphogenesis and Morphology

Reversible morphogenesis offers fungi a choice between two lifestyles within the host. When cells divide as yeast, a mother cell and her daughters remain confined at a single site and must compete with each other for nutrients. If nutrients are scarce, the formation of hyphae allows new cells to be produced sequentially by expansion at the tip. The bulk of the mother-cell cytosol, which contains most of the elements required to generate new cells, is pushed forward by turgor pressure coupled with expansion of the vacuoles positioned sub-apically [64]. Thus, the tip cell actively extends while the sub-apical cells lie dormant until new nutrients are assimilated. In *C. albicans*, each cell compartment is approximately 20 *μ*m long, so, after 5 cell divisions, the fungus can potentially cover a distance of 100 *μ*m. This is more than enough to escape from a phagocyte, anchor within a cell layer, or penetrate endothelia and reach the solid organs below. Once a more favourable environment is reached, the fungus can choose to revert once again to growth as yeast.

Morphogenesis from yeast or conidia is stimulated by a perceived change in the environment. For *A. fumigatus*, the signal for germination might be the presence of moisture in the lung. For *Malassezia*, it is thought to be the sensing of lipids. In *C. albicans*, morphogenesis has been studied extensively *in vitro*. It requires the integration of multiple signalling pathways involved in the sensing of ambient conditions, such as pH, temperature, and nitrogen availability (see reviews by [18, 19]). The complexity of these inputs reflects the unusually wide variety of host environments this fungus is capable of colonising. Signals act on the master regulators of morphogenesis, activators Cph1 and Efg1, and suppressors, Tup1 and Nrg1. The *efg1*Δ*/cph1*Δ double mutant has been widely used to study the role of morphogenesis in virulence and host responses because it does not produce hyphae neither does it express hypha-specific genes (HSGs) that are co-regulated with morphogenesis, making it difficult to pinpoint the key features of morphogenesis that are required for virulence. Early comparative expression studies revealed that several HSGs encode surface proteins that are involved in adhesion or host interactions and are essential for full virulence: Als3 is an adhesin and invasin, Hyr1 is involved in interaction with neutrophils, and Hwp1 delivers strong adhesion properties because it is a substrate for crosslinking to extracellular matrix by host transglutaminase [65, 66]. Other transcription factors that lie downstream of the Efg1/Cph1 master regulators have been identified as regulators of subsets of HSGs under defined *in vivo* conditions. These include Ume6, a master regulator of hypha-specific genes, Czf1 (embedded growth), Bcr1 (biofilm maturation), Eed1 (escape after endocytosis), and Hgc1, which suppresses cell separation and is expressed at the hyphal tip only [39, 67–72]. Analyses of temporal and spatial gene expression during infection, coupled with studies of physical changes induced by the environment in other fungi, suggest that a combination of site-specific and hypha-specific gene expression is likely to produce hyphae with subtly different properties [73, 74]. It is possible that some of the HSGs of unknown function are involved in modulating the structural status of hyphae. For example, *C. albicans* doubles the chitin content of the cell wall during hyphal growth, suggesting that increased rigidity is required, and other changes such as turgor pressure or the degree of cell wall crosslinking, could be important for the fungus to meet niche-specific challenges.

Morphogenesis has a significant consequence for the fungus because it exposes surface molecules that alert the immune system to its presence. The primary mechanism is through the detection of pathogen-associated molecular patterns (PAMPs), microbe-derived molecules that are recognised as nonhost by phagocytes. PAMPs that are derived from fungi include cell wall polysaccharides (galactomannan and galactofuranose in *A. fumigatus*; *β*-glucans, chitin phosphomannan in *C. albicans*), fungal surface proteins, secreted fungal enzymes and their breakdown products, and ATP released during host-cell lysis [31, 40, 75–77]. During epithelial colonisation by *C. albicans* and *Malassezia *yeasts, the fungal PAMPs that would otherwise induce an inflammatory response are masked from the immune system or simply not generated by the yeast form. *A. fumigatus* conidia are contained within a hydrophobic coat of RodA fibrils. Although taken up and cleared by macrophages, the inflammatory response is not activated unless conidia swell and germinate, when RodA is degraded and the cell wall polysaccharides are exposed [78, 79]. Mucosal defence at most body sites is mediated by epithelial cells and macrophages, which specifically recognise hyphae [80–82]. On detection of hyphal PAMPs via Dectin-1 and other receptors, cytokine signalling activates a group of proteins called the inflammasome, which is expressed within mucosal macrophages and dendritic cells [83]. The inflammasome processes and releases IL-1*β*, which recruits T cells and neutrophils, the key line of defence against *C. albicans* and *A. fumigatus* via phagocytosis and the deployment of neutrophil extracellular traps (NETs) (Figure 1(e)) [16, 84–86]. Thus, morphogenesis allows the fungus to be “seen” by the innate immune system so is only a virulence factor if host immunity is somehow defective.

### 3.2. Directionality

The hyphal tip is of particular importance because it controls the direction of new growth in response to the environment, steering the hypha around obstacles or towards nutrients [87]. In plants, the direction of polarised cell growth is determined by specific cues from the environment, such as light or gravity, which elicit pre-programmed directional responses, or tropisms. In fungi, contact-dependent growth behaviour, or thigmotropism, has been studied in some detail in plant pathogens. Growing hyphal tips are able to detect defined topographical features on the host leaf, enabling them to locate host penetration sites. For example, the hyphae of *Cymodothea trifolii *follow depressions at the cell boundaries of its host, white clover, because the stomatal pores are located at cell junctions [88]. A different growth strategy has evolved in *Uromyces appendiculatus*, a rust fungus whose host bean plant arranges its stomata transversely across the leaf. Here the hyphae grow perpendicularly to leaf depressions to maximise the chance of finding a penetration site. These responses can be induced on inert surfaces that topographically mimic the host, demonstrating that hyphae can sense surface topography through thigmotropism [89]. Are fungal tropic responses important for disease progression in humans? *A. fumigatus* has been termed “angiotropic” because its hyphae readily find their way into pulmonary blood vessels [90]. However, it is not clear whether they are actively responding to chemical or topographical clues and how this tropism is relieved when the fungus exits the bloodstream. The growth behaviour of *T. mentagrophytes* and *M. furfur* in the stratum corneum has been described as “meandering,” but this does not mean that the growth direction is random as it could be defined by local signals such as adhesion molecules. Other topical fungi, such as *T. violaceum*, *T. glabrum*, and *Microsporum gypseum*, specifically invade hair follicles, so are potentially following chemotropic gradients that define this site [91, 92]. Studies of tropisms in *C. albicans* and *C. dubliniensis* have shown that growth around small obstacles (thigmotropism) can be elicited in a calcium-dependent manner [87, 93–95]. However, no chemotropic growth has been observed in response to cAMP or retinoic acid, which can mediate growth behaviour in mammalian cells, although polarised mating projections do respond chemotropically to mating pheromone (Brand, unpublished data) [96]. Instead, pathogenic fungi seem to be hard-wired to penetrate any substrate they are in contact with, since hyphae in biofilms formed on inert silicones invade the abiotic material despite the apparent lack of extrinisic biological signals [7]. *C. albicans* responds to other external stimuli *in vitro*, displaying aerotropism under hypoxic conditions and galvanotropism, where hyphae germinate and orient towards the cathode in an applied electric field (Figure 2(c)(iii)) [94, 97, 98]. Galvanotropism is also calcium dependent in *C. albicans* and is a common feature of many types of polarised and migratory mammalian cells [99, 100]. These tropisms are potentially relevant to hyphal guidance within the host but are difficult to isolate in the complex *in vivo* environment. An exception is contact-dependent sinusoidal and helical growth, which occurs *in vivo* during onychomycosis when hyphae are embedded in the keratinised nail, and can be replicated *in vitro* in nutrient-poor, high-strength agar (4–6%) or on cellophane [36]. Helical growth is also observed in *Aspergillus *spp. that invade soft contact lenses and, therefore, could be a general hyphal response to embedding in a dense, dehydrated matrix [8]. While further tropic responses to *in vivo* stimuli have yet to be identified, normal regulation of hyphal tip directionality does seem to be involved in tissue penetration. In the *C. albicans rsr1Δ* mutant, where polarised growth is maintained but the direction of tip growth is erratic, hyphae are insensitive to all *in vitro* tropic stimuli (Figure 2(c)(iv)). Their ability to penetrate oral epithelial cells was reduced by 50%, and they were not able to cause cell damage compared to the control strain [42]. A refinement of this study confirmed that Rsr1 is required for damage to an epithelial monolayer but is not required for damage in deeper cell layers [44]. This suggests that a hyphal tip must orient correctly against the outer host surface to achieve initial cell penetration, but, once embedded, directional control is not required within a tissue matrix. This and other studies demonstrate that hyphal steering can be uncoupled from polarised growth, but there is no straightforward correlation between the loss of response to tropic stimuli that generate responses *in vitro* and avirulence *in vivo*. 

### 3.3. Force and Adhesion

Directional growth has to be coupled with mechanical force if hyphae are to push the surrounding matrix out of the way or to penetrate physical barriers. The hyphae of *A. fumigatus* and *C. albicans* are clearly capable of both feats within the host, yet little work on the biomechanics of tissue penetration in mammalian hosts has been carried out. More is known about the penetration of leaf cuticles by plant fungal pathogens. The waxy coating of the leaf is sufficiently tough to require the formation of specialised structures called appressoria, from which a penetration peg emerges to pierce the host leaf below. Two conditions must be in place for this to be successful. First, the appressorium must generate a high internal turgor pressure, which can be up to 5.85 MPa (58 atm) [101]. Secondly, the appressorium must become sufficiently anchored to the host leaf so that the penetration peg enters the plant rather than pushing the appressorium away from it. Thus, adequate adhesion to the host is required before the necessary pressure can be exerted by the fungus. This observation may explain why hyphae need to be so much stickier than yeast and why many of the genes upregulated during morphogenesis in *C. albicans* are related to adhesion. Although yeast cells adhere to the host perfectly well, their complement of adhesins may simply not be able to deliver the anchorage required during the application of hyphal tip pressure to an obstacle. The generation of sufficient adhesion by emerging hyphae may be a time-dependent process, where the number of crosslinks formed with the host increases with hypha length. This in turn could be influenced by whether the hypha is growing on a two-dimensional surface (low surface area of contact) or in a three-dimensional matrix (hypha surrounded by contact points). The need to establish multiple adhesion sites may be why active penetration of the host is not seen in the early stages of tissue invasion [44].

A further aid to applying force is turgor pressure, which must be accompanied by wall loosening at the tip so that the turgor pressure generated can be used maximally to apply tip pressure against an object [73]. The turgor pressure of hyphae has been determined in very few fungal species. In *Achlya bisexualis* and *Armillaria gallica* hyphae, it was found to be 0.6–0.8 MPa (6–8 atm), and the latter was able to apply a tip pressure of 17% of the turgor pressure against an object [73, 102]. In a rare study of force applied by a human pathogen, the oomycete *Pythium insidiosum*, it was calculated that the pressure generated by the hyphal tip was 0.3 MPa (3 atm). This was sufficient to drive a fungal tip through a stiffened 8% agar matrix (0.1 MPa or 1 atm) but was one hundred-fold less than that required to penetrate intact human skin [103]. Similar calculations have been undertaken for plant pathogens, and together the evidence indicates that host tissue penetration is likely to involve a combination of hyphal tip pressure and the hydrolytic activity of fungal exoenzymes [103, 104]. Turgor pressure and the secretion of wall tensioning and degradative enzymes must be coordinated at the site of growth to promote tissue penetration. Given the large number of hydrolytic enzymes that are secreted by hyphae, any study into which of them are involved in aiding tissue penetration, or whether their importance varies by body site, would require the isolation and analysis of apical-cell gene expression, which is no trivial task.

## 4. Future Perspectives

Although there are alternative routes to fungal virulence, many chronic and acute fungal infections involve the formation of hyphae during all or part of the infection process. The inbuilt propensity of hyphae to drill down into a substrate seems straightforward, but we are finding that many aspects of morphogenesis and hyphal growth behaviour are subtle and have yet to be explained. How are hyphal tips guided and controlled? What are the tropic mechanisms by which *A. fumigatus* enters and exits the bloodstream? How can tissue invasion by hyphae be separate from tissue damage? Are the structural properties of hyphae regulated differentially depending on body site? To understand how hyphae behave in the host environment, we will need to combine genetic approaches with cell physics. As the genome sequences and molecular tools become available in *Malassezia* and dermatophytes, about which we know relatively little, we should be able to generate a more global understanding of how hyphal growth is deployed against the host.
